# Evaluating the In Situ Effects of Whole Protein Coronas on the Biosensing of Antibody-Immobilized Nanoparticles Using Two-Color Fluorescence Nanoparticle Tracking Analysis

**DOI:** 10.3390/nano15030220

**Published:** 2025-01-30

**Authors:** Heeju Joung, Gwi Ju Jang, Ji Yeon Jeong, Goeun Lim, Sang Yun Han

**Affiliations:** Department of Chemistry, Gachon University, Seongnam 13120, Gyeonggi, Republic of Korea; 1997sara@naver.com (H.J.); jamiejang92@naver.com (G.J.J.);

**Keywords:** fluorescence nanoparticle tracking analysis, in situ protein coronas, biosensing, antibody-conjugated gold nanoparticles

## Abstract

The formation of protein coronas around engineered nanoparticles (ENPs) in biological environments is critical in nanomedicine, as these coronas significantly influence the biological behavior of ENPs. Despite extensive research on protein coronas, understanding the in situ influence of whole (soft plus hard) protein coronas has remained challenging. In this study, we demonstrate a strategy to assess the in situ effects of whole coronas on the model biosensing of anti-IgG using IgG-conjugated gold nanoparticles (IgG-AuNPs) through fluorescence nanoparticle tracking analysis (F-NTA), which enables the selective tracking of fluorescent particles within complex media. In our approach, anti-IgG and IgG-AuNPs were labeled with distinct fluorescent dyes. The accordance in hydrodynamic diameter distributions observed at two different wavelengths verifies the successful capture of anti-IgG on the IgG-AuNPs. The counting of fluorescent anti-IgG within the size distribution allows for a quantitative assessment of biosensing efficiency. This method was applied to evaluate the effects of four protein coronas—human serum albumin, high-density lipoproteins, immunoglobulin G, and fibrinogen—as well as their mixture across varying incubation times and concentrations. The results suggest that the physical presence of whole protein coronas surrounding the IgG-AuNPs may assist the biosensing interaction in situ rather than screening it.

## 1. Introduction

Engineered nanoparticles (ENPs) have found various applications in the field of nanomedicine, including diagnostics, therapy, and drug delivery [1,2,3,4,5]. When ENPs are exposed to biological environments, spontaneous formation of *protein corona* may occur immediately around the ENPs, potentially influencing their intended functions for therapeutic applications. Understanding protein coronas is therefore critical to nanomedicine, which has led to numerous investigations aimed at controlling protein coronas to develop effective nanomedicine over the past few decades [6,7,8,9,10,11].

The whole (full) protein corona that forms naturally in biological environments consists of two groups of proteins, namely *hard corona* (HC) and *soft corona* (SC). HC comprises proteins that tightly bind to ENPs through strong interactions. In contrast, SC consists of proteins that are weakly bound, possibly through protein–protein interactions with the HC, and primarily occupy the outer layer of the entire corona. These weakly bound proteins are susceptible to dynamic exchange with free proteins in the biological milieu as well [12,13,14].

Unlike HC, the subtle nature of SC and thus the whole corona has made it challenging to interrogate. For instance, common methods for isolating protein corona-coated ENPs from biological environments, such as high-speed centrifugation and size exclusion chromatography, often result in the loss of weakly bound SC proteins. As a result, most previous reports that investigated isolated protein corona–ENP complexes primarily focused on HC. Furthermore, SC is sensitive to environmental conditions; it can be released into the media or undergo time-dependent exchange with unbound proteins. Despite the challenges in investigating SC, it is essential to understand the whole protein corona (SC plus HC), particularly under in situ conditions, since this whole corona is what truly interacts with cells in biological systems. Unfortunately, the conventional analytical methods have not been optimally designed to probe the whole corona in situ, which include SC of a weakly bound and dynamic nature.

In recent years, significant efforts have been dedicated to developing strategies to investigate whole protein coronas. For example, cryo-electron microscopy (Cryo-EM) enables direct observation of the morphology of whole protein corona–ENP complexes in frozen media. Careful analyses using Cryo-EM, cryo-electron tomography (Cryo-ET), and statistical image analysis have provided insights into the protein–protein interactions involved in the corona and their association with ENP surfaces [15]. Additionally, the application of asymmetric flow field–flow fractionation (AF4) for the separation of corona–ENP complexes from blood plasma exhibited different protein patterns compared to those of HC obtained by isolation through centrifugation, as revealed by subsequent mass spectrometry (MS). This demonstrated a soft isolation method for whole coronas in distinct flow, enabling the characterization of weakly bound proteins [16]. The use of anti-PEG single-chain variable fragment (PEG-scFv)-based affinity chromatography (AfC) has also provided richer corona proteins than centrifugation, facilitating detailed analysis of SC protein compositions [17]. Furthermore, a unique strategy involving covalent capture of weakly bound proteins by HC proteins using in situ click chemistry has been presented, allowing for the MS characterization of SC proteins even after the isolation of corona–ENP complexes [18].

In addition, workflows integrating multi-parametric surface plasmon resonance (MP-SPR) and bio-layer interferometry (BLI) with MS have been proposed. These workflows provide a label-free assessment of the formation and kinetic changes in SC on ENPs immobilized on the biosensor surfaces [19,20]. Subsequently, MS is used for compositional analysis. In the MP-SPR study, a buffer solution was injected to wash off loosely bound proteins (SC), which was followed by the injection of surfactants to collect HC proteins from the surfaces of immobilized ENPs [19]. In the BLI study, different washing buffers were explored to collect corona proteins from biosensor tips, revealing discernable compositional differences attributed to SC and HC protein compositions [20]. Combined with downstream MS, these optical biosensing methods elucidated the kinetic evolution of SC proteins on ENPs immobilized on the biosensors. The use of magnetic beads to extract ENPs from blood serum allowed for fast affinity-based isolation of free-standing corona-coated lipid nanoparticles in the incubation solution, enabling further MS interrogation of associated corona proteins [21].

However, due to the delicate nature of SC, it remains more suitable to investigate the whole corona–ENP complexes under in situ conditions, i.e., in their native incubation media. To this end, various spectroscopic methods such as fluorescence correlation spectroscopy (FCS) and anisotropy-resolved multi-dimensional emission spectroscopy (ARMES) have been demonstrated to facilitate less perturbative in situ probing of protein interactions with ENPs [22,23]. In addition to these in situ ensemble measurements, 3D real-time single-particle tracking spectroscopy has been demonstrated for in situ particle-by-particle characterization of protein coronas [24].

Diffusion-based techniques, such as dynamic light scattering (DLS) and nanoparticle tracking analysis (NTA), have been widely used to characterize ENPs by measuring the hydrodynamic diameters (*d*_h_) and zeta potentials of ENPs, which allowed for characterization of corona growth around them [25,26,27]. DLS measures the autocorrelation function of scattered lights in the time domain, which reflects the diffusive motion of ENPs in suspension, whereas NTA tracks individual particle motions in video sequences over time. Both methods determine nanoparticle size distribution in *d*_h_ through the Stokes–Einstein equation. DLS measurements may be affected by other particulates such as protein aggregates present in the solution, which thus needs pure samples. In contrast, NTA’s ability to track individual particles allows for reliable measurements of particle number density and polydispersity, as well as easy implementation of fluorescence NTA (F-NTA). F-NTA advantageously detects fluorescent ENPs against background particulates, which was successfully applied in characterizing exosomes [28,29,30] and in situ monitoring of the formation of intact whole protein corona around ENPs in its native environment recently [29,30,31,32]. Although these methods have been successfully demonstrated for characterizing the formation of whole protein corona on ENPs, knowledge on the influence of the whole coronas, which alters the biological efficacy of ENPs, remains elusive.

Herein, we demonstrate that two-color F-NTA offers a facile strategy for assessing the impact of in situ protein coronas on the biosensing capabilities of IgG-immobilized nanoparticles. As a model for target-oriented nanomedicine, IgG-conjugated PEGylated gold nanoparticles (IgG-AuNPs) were utilized [33,34,35]. Utilizing the unique advantages of F-NTA, specifically its ability to assess successful capture through correlating binding events with size distributions and quantifying fluorescent particles within complex media, we investigated the in situ effects of whole protein coronas on the affinity capture of anti-IgG by IgG-AuNPs. In the study, the influences of individual protein coronas were explored, which included the protein coronas of human serum albumin (HSA), high-density lipoproteins (HDL)s, fibrinogen (Fib), as well as their mixture (Mix), across various incubation times and concentrations. The results demonstrated that F-NTA, a technique widely available in common nanomaterial laboratories, provides a straightforward opportunity to elucidate the impacts of in situ protein coronas on the functional performance of ENPs.

## 2. Materials and Methods

### 2.1. Materials

IgG (from mouse serum) and anti-IgG (F_ab_ specific, mouse) antibodies produced in goats, citrated AuNP, HSA, IgG (from human serum), Fib, human serum, sodium bicarbonate, MES (2-(*N*-morpholino)ethanesulfonic acid), and PBS (phosphate-buffered saline) buffers were commercially obtained from Sigma-Aldrich Co. (St. Louis, MO, USA). In addition, 2k SH-PEG-COOH and 1k mPEG-SH were purchased from Biochempeg PEG Scientific Inc. (Watertown, MA, USA). Cy5-NHS (*N*-hydroxysuccinimide) ester was purchased from Cytiva (Malborough, MA, USA). Alexa Fluor 488-NHS ester, Zeba spin desalting column, EDC (1-Ethyl-3-(3-dimethylaminopropyl)carbodiimide) and Sulfo-NHS were obtained from Thermo Fisher Scientific Inc. (Waltham, MA, USA). HDL was purchased from Sigma-Aldrich Co.

### 2.2. Preparation of Fluorescently Labeled IgG and Anti-IgG

A 5 µL aliquot of Cy5-NHS ester (2 mg/mL in dimethylformamide (DMF)) was added to 50 µL of a freshly prepared IgG solution (2 mg/mL in 0.1 M sodium bicarbonate). The mixture was stirred for 10 min at room temperature. The labeled IgG was isolated and purified using a Zeba spin desalting column using PBS buffer serving as the eluent. The labeling procedure for anti-IgG was similar; however, Alexa Fluor 488-NHS ester (2 mg/mL in DMF) was used as the labeling reagent, and the solution was stirred for 2 h at room temperature.

### 2.3. Preparation of IgG-AuNPs

A 30 µL aliquot of mixed PEGylation solution (2k SH-PEG-COOH:1k *m*PEG-SH = 1:9 in DW, total 1 mM) was added to 1 mL citrated AuNPs (6.5 × 10^11^ particles/mL). The mixture was centrifuged at 9000 rpm (7634× *g*) for 30 min with deionized water, and the supernatant was removed. The addition of the PEGylation solution and the washing procedure via centrifugation were repeated three times. Then, 100 µL of EDC (12 mM in MES buffer) and 100 µL of sulfo-NHS (60 mM in MES buffer) were added to the resulting PEGylated AuNPs. Then, 10 µL of Cy5-labeled IgG was mixed into the solution, which was stirred for 5 h at room temperature. To remove unbound IgG, the solution underwent centrifugation at 9000 rpm at 4 °C for 30 min with PBS, and the supernatant was discarded. This step was repeated three times, and the PBS buffer was added to the pellet to achieve a final volume of 200 µL.

### 2.4. Incubation of Protein Coronas and Biosensing of Anti-IgG

Protein coronas of HSA, HDL, IgG (human), Fib, and their mixture were incubated around Cy5-derivatized mouse IgG-conjugated PEGylated AuNPs (IgG-AuNPs) in a shaking incubator at 400 rpm at 37 °C for varying incubation periods (from 1 h to 12 h). At each incubation period, 10 µL of Alexa Fluor 488-labeled anti-IgG was added to the incubation solution and allowed to incubate at 400 rpm at 37 °C for 2 h for biosensing. To remove unbound anti-IgG and proteins, the solution was centrifuged at 9000 rpm at 4 °C for 30 min with PBS buffer, and the supernatant was discarded. The removal procedure was repeated three times.

### 2.5. NTA Measurement

ENPs in the solution were characterized using a Nanoparticles Tracking Analyzer (PMX-430 QUATT, Particle Metrix GmbH, Meerbusch, Germany). The NTA system was equipped with four lasers of different wavelengths (405 nm, 488 nm, 520 nm, and 640 nm) and CMOS camera sensors for fluorescence measurements of particles in suspension. In this study, the 488 and 640 nm lasers were utilized for fluorescence excitation. Prior to each measurement, a cell check was conducted by aligning the camera and laser using standard 100 nm polystyrene beads and optimizing the profile auto-symmetry. The samples were diluted in 1× PBS buffer to achieve an optimal particle count per frame in fluorescence mode targeting 150–200 particles. Measurements were performed at 11 distinct positions within the cell with typical camera parameters: sensitivity 85, shutter 100, minimal area 20, maximal area 1000, brightness 20, and temperature of 22 °C. For fluorescence measurements, the laser cut-off filter at 500 nm was employed when the 488 nm laser served as the excitation source, and the filter at 660 nm was used for the 640 nm laser. To minimize fluorescence bleaching, the sample was displaced by a volume of 20 µL after each photograph was taken. For each photograph, three cycles per frame were taken. Measurements were repeated until a data set comprising at least 10,000 particles was accumulated.

### 2.6. Characterization by UV–VIS Spectrophotometry and TEM

NP samples were characterized using a UV–VIS spectrometer (Cary 5000, Agilent Technologies, Inc., Santa Clara, CA, USA) across a wavelength range of 200 nm to 800 nm at room temperature. Typical parameters included an average time of 0.100 s, a data interval of 0.1 nm, and a scan rate of 60.00 nm/min. For TEM imaging, a transmission electron microscope (Tecnai G2 F30, FEI; 300 kV, Thermo Fisher Scientific Inc., (Waltham, MA, USA)) at the Center for Bionano Materials Research (Gachon University, Republic of Korea) was utilized.

## 3. Data and Results

### 3.1. Assement Strategy

**Figure 1:**
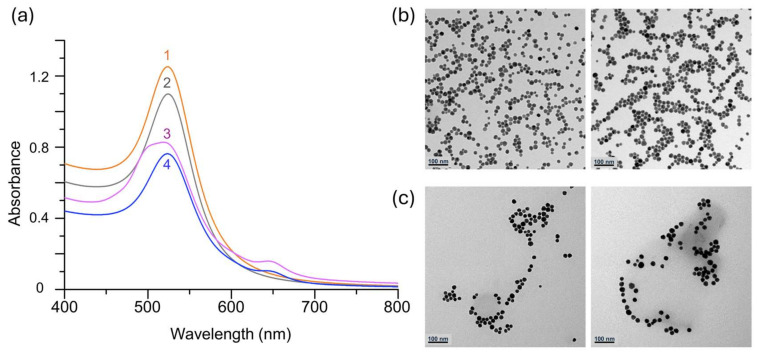
(**a**) UV–VIS spectra of 1. citrated AuNPs, 2. PEGylated AuNPs, 3. IgG-AuNPs, and 4. anti-IgG; TEM images of (**b**) PEGylated AuNPs and (**c**) anti-IgG@IgG-AuNPs.

In this study, we employed the immune-capture of anti-IgG (F_ab_ specific, mouse) using IgG (mouse)-AuNPs as a model system to examine the in situ influence of whole protein coronas produced around the IgG-AuNPs. IgG-AuNPs were synthesized through ligand exchange of citrate-stabilized AuNPs with a mixed thiol PEGylation reagent consisting of 2k SH-PEG-COOH and 1k *m*PEG-SH in a ratio of 1:9. The longer 2k PEG reagent, possessing a reactive carboxyl (-COOH) terminal, was exploited to covalently attach to Cy5-labeled IgG via NHS/EDC chemistry (Figure 1, characterization). The resulting IgG-AuNPs possessed PEGylated surfaces designed to minimize non-specific adsorption on the NP surfaces, for which F-NTA estimated that the anti-IgG on the surfaces was very small, i.e., less than 1% of total PEGylated AuNPs, in the IgG solution.

The use of fluorescence labeling (Cy5) on IgG enabled the tracking of IgG-conjugated AuNPs through the characteristic fluorescence at λ_ex_ = 640 nm. This fluorescence allowed the IgG-AuNPs to be distinguished from background components such as non-fluorescent protein aggregates, which could otherwise interfere with the measurement such as size distribution in *d*_h_ under the in situ conditions. The anti-IgG used to evaluate the efficacy of biosensing was also labeled, but with a distinct dye (Alexa Fluor 488, λ_ex_ = 488 nm).

**Figure 2:**
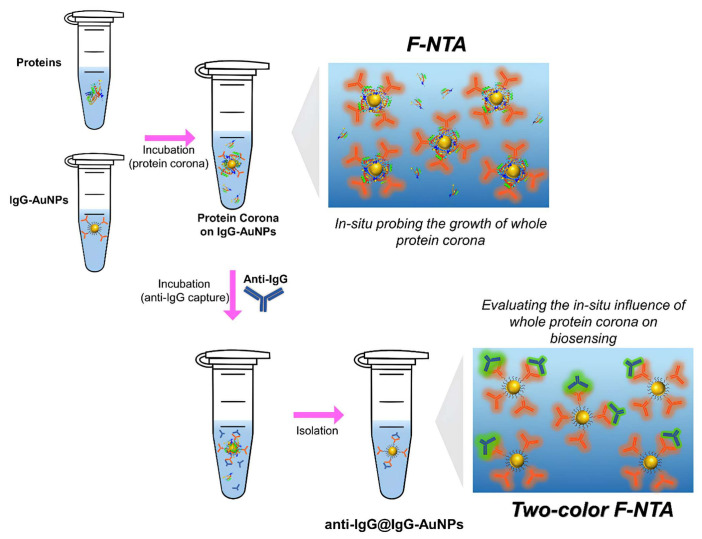
Experimental scheme for in situ monitoring of whole corona growth using F-NTA at 488 nm and assessing the in situ influence of whole protein coronas on the biosensing efficiency.

When the fluorescence-labeled anti-IgG was affinity-captured on IgG-AuNPs, the complexes of anti-IgG@IgG-AuNPs were formed. For these complexes, F-NTA at the excitation wavelength for anti-IgG (488 nm) produced a size distribution consistent with that obtained at λ_ex_ for IgG-AuNPs (640 nm). This consistency verifies the formation of anti-IgG@IgG-AuNPs complexes. In other words, this agreement in size distributions confirmed that the biosensing of anti-IgG truly occurred on IgG-AuNPs (Figure 2). Moreover, counting the number of particles (the number density) within the size distribution of the complexes, which are detected in F-NTA at λ_ex_ for anti-IgG, allows for quantitative monitoring of the biosensing efficacy of anti-IgG on IgG-AuNPs. Thus, the two-color F-NTA method provides a facile means to assess the biosensing efficiency of ENPs under various in situ conditions, where binding is validated by the observed accordance in size distributions from the two respective λ_ex_s.

In basic terms, this strategy relies on the capability of F-NTA to discern fluorescent ENPs in complex environments. However, in this method, to address practical challenges, additional steps were incorporated. Firstly, as the study examined the effects of incubation time for corona formation over a prolonged period up to 12 h, certain nanoparticle aggregation was unavoidable. To mitigate this issue, the *d*_h_s of IgG-AuNPs was measured separately after incubation in the PBS solution (1×) for the same duration as the protein corona incubation. The measured *d*_h_s of protein corona-coated IgG-AuNPs, which was incubated in the protein solution, was adjusted against those for IgG-AuNPs in PBS after the same incubation period. The results were expressed as Δ*d*_h_, calculated as *d*_h_(IgG-AuNPs in the protein solution) − *d*_h_(IgG-AuNPs in PBS), which reflects the size increase caused by in situ corona formation.

Secondly, to reduce background interference, after the incubation for biosensing, ENPs including unbound IgG-AuNPs and anti-IgG@IgG-AuNPs complexes were isolated by high-speed centrifugation. The size distribution in *d*_h_ of the ENPs after being resuspended in PBS was then analyzed by F-NTA at the two λ_ex_s. In fact, it was found that this step was essential to overcome the significant fluorescence background from unbound Cy5-labeled anti-IgG in the sample, which complicated F-NTA measurements. Although the measurements were conducted on the ENPs isolated from the incubation solution, the immunobinding between anti-IgG and IgG-AuNPs might be sufficiently strong to survive the isolation process just like in the process commonly utilized in the F-NTA characterization of exosomes [28,29,30]. Consequently, it can be concluded that even after separation from the incubation environment, the quantitative data regarding the extent of biosensing under in situ conditions were largely preserved. The isolation process also assists in eliminating unwanted anti-IgG that may have been non-specifically adsorbed by the ENP surfaces. Thus, the application of two-color F-NTA enables the monitoring of the in situ growth of whole coronas and evaluating their influence on the biosensing capabilities of ENPs.

### 3.2. In Situ Time-Dependent Growth of Whole Protein Coronas on IgG-AuNPs Probed by F-NTA

**Figure 3:**
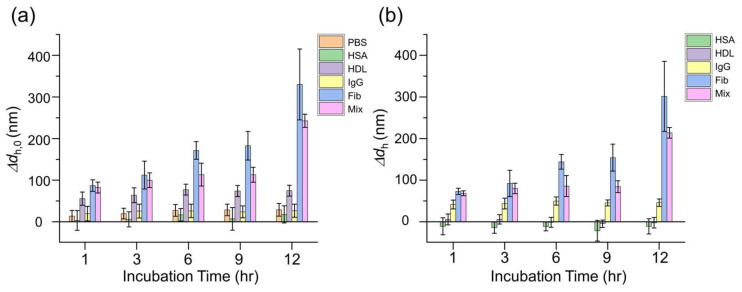
In situ growth of whole protein coronas around IgG-AuNPs, with hydrodynamic sizes measured at λ_ex_ = 640 nm in the respective incubation solutions of HSA, HDL, IgG, Fib, and Mix over the incubation periods of up to 12 h. PBS refers to the incubation in PBS without proteins. (**a**) Δ*d*_h,0_ is the adjusted value for *d*_h_ in PBS at 0 h and (**b**) Δ*d*_h_ is the adjusted value for *d*_h_ in PBS at the corresponding incubation time.

F-NTA is well suited for probing the in situ growth of whole protein coronas within complex environments [31,32]. In our study, we first investigated the in situ corona on IgG-AuNPs in the respective protein solutions using F-NTA at λ_ex_ = 640 nm (Video S1 in Supplementary Materials). The following protein coronas of four representative serum proteins and their mixture were examined: HSA (5–7 nm in diameter, 40 mg/mL in human serum in average), IgG (human serum, 10–15 nm, 10 mg/mL), Fib (45 nm, 2 mg/mL) and HDL (7–14 nm, 0.6 mg/mL), which are biomolecular complexes of apolipoproteins and lipids. Mix was prepared to maintain each protein at the same concentration level used in the respective studies. Those proteins were frequently found in previous studies for serum protein coronas as well. The time-dependent growth of whole protein coronas was assessed in terms of the *d*_h_ of IgG-AuNPs in each protein solution, which was diluted to 1/10 of their average concentrations in human serum, over incubation periods of up to 12 h. After each incubation period, F-NTA was performed directly on the incubation solutions to ensure the in situ conditions. The results are presented in Δ*d*_h_, which represents the *d*_h_ of whole protein corona-coated IgG-AuNPs corrected for particle aggregation. The analysis was performed in two ways: one method corrected the *d*_h_ of IgG-AuNPs incubated in PBS without further incubation (Δ *d*_h_,_0_, at 0 h) and the other was adjusted for that in PBS after the same incubation duration for corona formation (Δ*d*_h_). In Figure 3a, the aggregation of IgG-AuNPs was evident as shown in the increase in ENP size in PBS. Thus, the adjusted Δ*d*_h_ for the *d*_h_ of IgG-AuNPs in PBS (Figure 3a) for the corresponding incubation time was regarded to be the size variation due to the protein corona growth on IgG-AuNPs (Figure 3b).

Figure 3b displays the time-dependent variations in the hydrodynamic size (Δ*d*_h_) of IgG-AuNPs in respective protein solutions, revealing the in situ growth of whole protein coronas around the IgG-AuNPs. The results showed that the sizes of whole protein coronas generally increased gradually as the incubation time elapsed (Figure 3a). For example, the whole protein corona of Fib gradually increased, which significantly increased the *d*_h_ of ENPs by about 300 nm (Δ*d*_h_) following a 12 h incubation period. Notably, despite different respective concentrations in the incubation solutions (Mix > HSA > IgG > Fib > HDL), the order of whole corona growth was found to be Fib > Mix > IgG > PBS (no proteins) > HDL > HSA (Figure 3b). It suggests that the sizes of whole protein coronas are more closely related to protein sizes than to their concentrations.

Intriguingly, the size behaviors due to the formation of HSA and HDL coronas were particularly notable (Figure 3b). The PBS condition, which involved the incubation of IgG-AuNPs without proteins in PBS, showed a size increase of more than 10 nm, presumably due to the aggregation of IgG-AuNPs (Figure 3a). However, the protein adsorption of HSA and HDL resulted in negative size changes, suggesting that their presence on ENP surfaces helps prevent ENP aggregation. Previous studies have indicated that the involvement of small proteins such as HSA in coronas could reduce nanoparticle aggregation [36,37]. Although HDL is relatively large as intact clusters, it has been suggested that these clusters disintegrate into individual molecules on nanoparticle surfaces upon adsorption and subsequent surface coating [38,39]. Thus, the observed negativeness in the growths of HSA and HDL coronas indicates that their presence on the IgG-AuNP surfaces contributes to reduced aggregation of IgG-AuNPs. Overall, the *d*_h_s of the protein corona-coated IgG-AuNPs measured by F-NTA enabled the observation of in situ whole corona growth over time. The findings suggest that the protein size is more important in determining the resultant size of whole coronas than protein concentration, whereas the presence of small proteins like HSA presumably mitigates nanoparticle aggregation.

### 3.3. Assessment of the In Situ Influence of Whole Protein Coronas on the Biosensing of Anti-IgG Using Two-Color F-NTA

In the previous section, the in situ growth of whole protein coronas around IgG-AuNPs was investigated by measuring time-dependent variations in Δ*d*_h_ using F-NTA directly in the incubation solution. However, the evaluation of the influence of whole coronas on biosensing employed a different approach. In this approach, whole protein coronas were incubated on IgG-AuNPs with proteins at varying concentrations over periods of up to 12 h. After the corona incubation, anti-IgG was added and affinity-captured by IgG-AuNPs in the presence of the whole protein coronas, which resulted in the formation of anti-IgG@IgG-AuNP complexes. After the incubation, the ENPs were isolated from the incubation solution by high-speed centrifugation and transferred to a PBS solution, where F-NTA measurements were conducted at two λ_ex_s of 640 nm and 488 nm to track the ENPs according to IgG-AuNPs and anti-IgG, respectively (Figure 2). Unlike the F-NTA employed in the in situ growth study, which was performed directly on the incubation solution at 640 nm, the biosensing study faced significant background fluorescence from free anti-IgG in the solution, which greatly interfered with fluorescence tracking. Therefore, the ENPs were isolated from the incubation solution. It was presumed that the affinity binding of anti-IgG to the IgG-AuNPs was sufficiently strong to withstand the washing process. In this regard, the obtained results were considered to preserve the biosensing events that occurred in the in situ conditions, although the final measurements were performed in PBS after isolation of ENPs.

**Figure 4:**
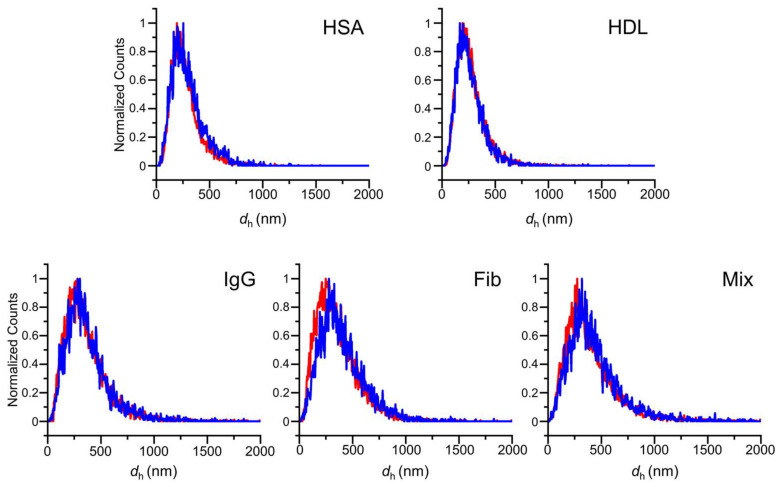
The comparisons of the size distributions in *d*_h_ of isolated ENPs at two λ_ex_ of 488 nm (red) and 640 nm (blue) after 6 h incubation in the respective protein solutions of HSA, HDL, IgG, Fib, and Mix.

F-NTA measurements were conducted on the isolated ENPs at λ_ex_ of 640 nm and 488 nm after incubation for periods of up to 12 h. The excitation at 640 nm tracked the fluorescence of IgG immobilized on the AuNPs, thus representing the IgG-AuNP particles. The 488 nm excitation tracked the fluorescence of anti-IgG, which would display the same size distribution as that obtained at 640 nm, provided that the anti-IgG is bound to the IgG-AuNPs. Thus, the accordance between the two distributions at the different excitation wavelengths assures that the measurements is indeed for anti-IgG@IgG-AuNPs, which indicates successful biosensing of anti-IgG using the IgG-AuNPs (Figure 4) (Videos S2 and S3 in Supplementary Materials). In addition, F-NTA measures the size distribution of particles and also counts those particles (fluorescent particles/mL) that are associated with the measured size distribution. In other words, the background fluorescence from unbound anti-IgG in the solution was excluded from the counts in *d*_h_ measurement. Thus, the counting of fluorescent particles at 488 nm provides insight into the quantity of anti-IgG that is captured on the surfaces of IgG-AuNPs only.

**Figure 5:**
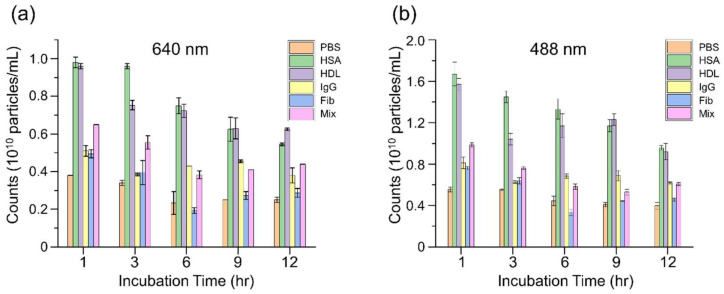
Time-dependent variations in the particle counts for isolated ENPs incubated in the respective incubation solutions (1/10) of HSA, HDL, IgG, Fib, and Mix measured at (**a**) 640 nm and (**b**) 488 nm. PBS refers to the incubation in PBS without proteins.

Figure 5 presents the number of isolated particles counted at 640 nm and 488 nm over the incubation period of up to 12 h. The apparent correspondence between the time-dependent data at the two wavelengths also indicates that the measurements were indeed reflective of anti-IgG@IgG-AuNP complexes. In the results, the number of IgG-AuNP particles counted at 640 nm tended to decrease over time, which we speculate may be attributable to experimental factors such as particle precipitation as the incubation time increased. However, the decrease in particles did not affect the assessment of biosensing efficiency of this study, as the efficiency was evaluated by comparing the counts of anti-IgG@IgG-AuNPs at 488 nm with those of IgG-AuNPs at 640 nm. The similar results over time obtained at both 640 nm and 488 nm indicate that the measured particles are indeed the complexes of anti-IgG-captured by IgG-AuNPs, i.e., anti-IgG@IgG-AuNP. This approach thus provides a method that is applicable to assess the biosensing efficiency reflecting various influences of in situ environments such as whole protein coronas.

**Figure 6:**
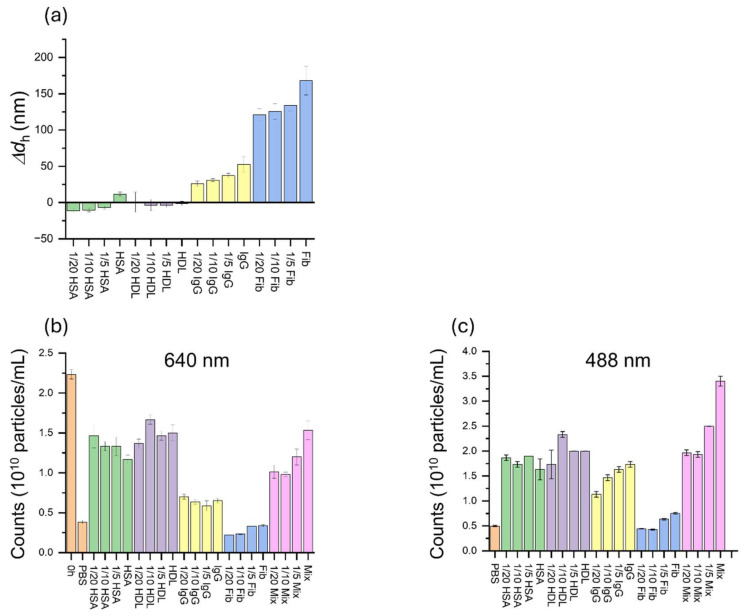
Concentration dependence according to (**a**) the size of whole protein coronas in the incubation solution measured by F-NTA at 488 nm, and the particle counts for isolated ENPs incubated in the respective protein solutions of HSA, HDL, IgG, Fib, and Mix. These are measured over a range of protein concentrations from 1/20 to 1 of average concentrations in the serum after 6 h incubation, (**b**) λ_ex_ = 640 nm and (**c**) 488 nm.

Finally, using the method described above, we explored the concentration dependence of biosensing on protein concentration used in the incubation. In this study, four proteins and their mixture were examined over a concentration range from 1/20 to 1 of the respective average concentrations found in human serum with a fixed incubation period of 6 h. The concentration dependence of the sizes of in situ whole coronas was investigated in the incubation solution by F-NTA. As shown in Figure 6a, the sizes of the whole protein coronas increased with higher protein concentrations in the solution.

In the following two-color F-NTA study on isolated ENPs after further incubation with anti-IgG, the agreement of data obtained at the two excitation wavelengths confirmed that the experimental procedure was well executed and the counting at 488 nm (for anti-IgG) was indeed performed on the successful capture of anti-IgG by IgG-AuNPs, specifically anti-IgG@IgG-AuNP complexes (Figure 6b,c). As found in the results obtained at 640 nm (for IgG-AuNPs), the number of IgG-AuNPs may vary during the experimental process. However, this variability can be adjusted by normalizing the 488 nm results (for anti-IgG) using the 640 nm results (for IgG-AuNPs), which facilitated the assessment of binding efficiency.

**Figure 7:**
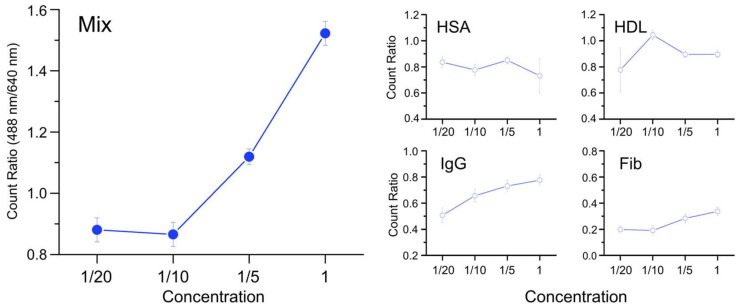
Concentration dependence of the binding efficiencies of anti-IgG to IgG-AuNPs evaluated by count ratio (488 nm/640 nm), which were incubated in the respective protein solutions of HSA, HDL, IgG, Fib, and Mix for 6 h.

Figure 7 displays the normalized results for 488 nm relative to 640 nm, which corresponds to the normalization of the number of captured anti-IgG with respect to the number of IgG-AuNPs involved in the assessment. In fact, the fluorescence quantum yields for the two dyes are different so the ratio may not yield the information on the absolute number ratio. In the results, small proteins such as HSA and HDL did not show an apparent propensity for concentration dependence. This lack of dependence may be associated with their complex behaviors in the coronas, such as involvement in reducing ENP aggregation. However, larger proteins including IgG and Fib exhibited a concentration dependence as their concentrations increased for incubation and the biosensing efficiency also increased. This finding suggests that the presence of whole protein coronas on the surfaces of IgG-AuNPs may promote the bio-interaction between IgG on the AuNPs and anti-IgG in the environment rather than interfering with it, despite the fact that the thickness of whole protein coronas increased at a higher incubation concentration (Figure 6a). It is more evident in the case of Mix, which exhibited a significant increase in biosensing efficiency. This may be attributed to an improved orientation of surface-bound IgG towards anti-IgG in the solution, which is facilitated by the physical presence of adsorbed proteins, or whole coronas, on the surfaces of ENPs. This phenomenon is analogous to the use of protein A or PEGylated self-assembled monolayers in the surface immobilization of antibodies on ENPs, which facilitates the outward orientation of antibodies on surfaces, promoting favorable protein–protein interactions with unbound targets in the solution [40].

## 4. Conclusions

Understanding the impact of whole protein coronas (soft plus hard) on the designed performance of ENPs in in situ environments remained challenging due to lack of suitable analytical methods. In this study, we demonstrated a simple two-color F-NTA approach for evaluating the in situ effects of whole protein coronas on biosensing, for which the capture of anti-IgG on IgG-AuNPs was employed as a model system. This method exploited the unique advantages of F-NTA, which include the capability of measuring the distribution of *d*_h_s of fluorescent particles in the complex media in a quantitative way. By adopting the two-color method, the successful capture of anti-IgG using IgG-AuNPs, which were labeled with distinct fluorescent dyes with λ_ex_ = 488 nm and 640 nm, respectively, was confirmed by the accordance in observed size distributions at the two λ_ex_s. It arose from the resulting complexes of anti-IgG@IgG-AuNPs. In addition, the counting of particles, which emit fluorescence of anti-IgG that is bound to IgG-AuNPs (488 nm), provided quantitative information on the biosensing efficiency with normalization to that of particles with fluorescence from IgG-AuNPs (640 nm).

In the study, the whole protein coronas of HSA, HDL, IgG, Fib, and Mix were examined. In particular, the measurements of *d*_h_ using F-NTA revealed the in situ growth of whole protein coronas in the incubation solution over a time range up to 12 h. In the results, the protein coronas grew larger as the incubation time increased. The adjustment for nanoparticle aggregation revealed that the presence of coronas of small proteins such as HSA and HDL on the ENP surfaces mitigated the aggregation of ENPs. Using the two-color F-NTA strategy, the biosensing efficiency was evaluated after isolation of ENPs from the incubation solution, in which the in situ binding of anti-IgG to IgG-AuNPs was presumably preserved. We successfully evaluated the influence of those protein coronas around IgG-AuNPs on the affinity capture of anti-IgG across varying incubation times and concentrations. Importantly, it was revealed that the presence of in situ protein coronas on the ENP surfaces promotes the biosensing interaction of surface-conjugated IgG with anti-IgG rather than screening the interaction. Therefore, it can be suggested that it is the biochemical identity of corona proteins rather than their physical presence that more importantly determines the biological fates of ENPs.

Accordingly, it is demonstrated in this study that two-color F-NTA experiments offer a valuable opportunity to investigate the influence of whole protein coronas under in situ conditions, which includes probing the growth of protein coronas as well as assessing their impact on the biosensing efficiency of ENPs.

## Data Availability

The data presented in this study are available from the corresponding author upon request.

## Supplementary Materials

The following supporting information can be downloaded at https://www.mdpi.com/article/10.3390/nano15030220/s1, Video S1: NTA of whole HSA corona at 640 nm and Video S2: NTA of anti-IgG bound HAS-corona IgG AuNPs at 488 nm and Video S3: NTA of anti-IgG bound HSA corona-IgG AuNPs at 640 nm.

## Abbreviations

The following abbreviations are used in this manuscript:

ENPs	Engineered nanoparticles
IgG-AuNPs	IgG (mouse)-conjugated PEGylated gold nanoparticles
F-NTA	Fluorescence nanoparticle tracking analysis
HC	Hard corona
SC	Soft corona
Cryo-EM	Cryo-electron microscopy
Cryo-ET	Cryo-electron tomography
AF4	Asymmetric flow field-flow fractionation
MS	Mass spectrometry
PEG-scFv	PEG single-chain variable fragment
AfC	Affinity chromatography
MP-SPR	Multi-parametric surface plasmon resonance
BLI	Bio-layer interferometry
FCS	Fluorescence correlation spectroscopy
ARMES	Anisotropy-resolved multi-dimensional emission spectroscopy
DLS	Dynamic light scattering
NTA	Nanoparticle tracking analysis
HSA	Human serum albumin
HDL	High-density lipoproteins
IgG	IgG (human)
Fib	Fibrinogen
Mix	Mixture of HSA, HDL, IgG, and Fib
anti-IgG	Anti-IgG (mouse)
